# A221 AZATHIOPRINE DOSING THRESHOLD IN ANTI-TNF COMBINATION THERAPY FOR MINIMIZING IMMUNOGENICITY AND OPTIMIZING TREATMENT EFFICACY FOR PATIENTS WITH INFLAMMATORY BOWEL DISEASE. A RETROSPECTIVE COHORT STUDY

**DOI:** 10.1093/jcag/gwad061.221

**Published:** 2024-02-14

**Authors:** N Alajeel, J Choi, R Khanna, A Wilson

**Affiliations:** Internal Medicine, London Health Sciences Centre, London, ON, Canada; Internal Medicine, London Health Sciences Centre, London, ON, Canada; Internal Medicine, London Health Sciences Centre, London, ON, Canada; Internal Medicine, London Health Sciences Centre, London, ON, Canada

## Abstract

**Background:**

Reported rates of anti-drug antibody (ADA) formation to tumor necrosis factor-a antagonists (anti-TNF) range from 20-75%. One way to reduce the risk of ADA formation is to combine a second immune-suppressing agent such as azathioprine (AZA) with the anti-TNF agent, known as combination therapy. However, it is unknown if the risk of combination therapy, including the additive risk of AZA and anti-TNF side effects, could be reduced by exposing individuals with inflammatory bowel disease (IBD) to the lowest dose of azathioprine while still maintaining the beneficial effect of ADA prevention.

**Aims:**

To identify the minimum dose of AZA needed to prevent ADA formation to anti-TNF agents in individuals with IBD receiving combination therapy. We also assessed the occurrence of adverse drug events, treatment loss of response, treatment discontinuation and the need for treatment dose escalation.

**Methods:**

A retrospective cohort study is ongoing in adult participants with IBDreceiving either infliximab or adalimumab in combination with AZA. Patients are divided based on their AZA dosage (low dose group, AZA ampersand:003C2mg/kg/day versus standard dose group, AZA 2mg/kg/day). All participants will be followed for 1 year and observed for the occurrence of ADA formation, adverse drug events, treatment loss of response, treatment discontinuation and the need for treatment dose escalation.

**Results:**

To date, 48 participants are currently included (low dose, n=29; standard dose, n=19). 42 are on Infliximab and 6 are on Adalimumab. The occurrence of ADA was 17.4% in the low dose group and 20% in the standard dose group (pampersand:003E0.99). More participants lost response to treatment and discontinued anti-TNF therapy in the standard dose group (n=7/19, 36.8%) versus the low-dose group (n=8/29, 27.6%, p=0.54). More participants in the low-dose group received anti-TNF dose escalation (n= 20/29,68.9%) compared to the standard dose group (n=6/19, 31.6%, p=0.017). Adverse events, to Anti-TNF was seen in (n=3/19, 15.7%) in the standard dose group, in comparison to (n=4/29, 13.79%, pampersand:003E0.99) in the low dose group. Adverse events to AZA were surprisingly higher in the low dose group (n=14/29, 48.3%) while in the standard dose, it was (n=5/19, 26.3%, p=0.14).

**Conclusions:**

The preliminary result of the study suggests that there is no significant difference in anti-drug antibody formation between standard and low doses of azathioprine in combination with anti-TNF therapy, in addition, data showed significant lower rates of Anti-TNF discontinuation and loss of response to treatment in low Azathioprine dose group. Completion of the study will help further define if low-dose AZA can be used for ADA prevention in anti-TNF combination therapy without compromising important clinical outcomes.

**Funding Agencies:**

None

